# Meetings of Societies

**Published:** 1923-01

**Authors:** 


### MEETINGS OF SOCIETIES.

### Bristol Medico-Chirurgical Society.

*November 8th*, 1922.

J. A. Nixon, C.M.G., M.D., F.R.C.P., President, in the Chair.

Mr. Forbes Fraser (Bath) read a paper on "**Some Points in the Surgical Treatment of the Peritoneum**" (*vide* pp. 29 to 41).

The President hoped that the modern student did not neglect to read that great classic Hilton's *Rest and Pain*. He asked why it was considered good to leave effusion in the peritoneal cavity whereas the pleura looked after itself better when left dry. He thought in pneumococcal peritonitis convalescence was shortened by evacuating the fluid early.

Mr. Lansdown described the routine treatment of peritonitis at Guy's Hospital in his student days. He agreed with the lines of treatment advocated by Mr. Fraser.

Mr. Walters considered the mortality rate had been greatly reduced since the introduction of treatment in the Fowler position and by rectal saline injections. The class of case in which there was present a collection of foul fluid, dark in colour, like dish water, required drainage. He gave morphia the first night and later used aspirin.

Mr. Carwardine always drained a case of perforated stomach or duodenal ulcer, usually with several tubes. He had published a series of 13 consecutive cases with 12 recoveries.

Mr. Warren thought pelvic drainage might allow escape of fluid from other parts of the peritoneal cavity. The effusion soon lost its protective power. Exercise had to be considered as well as rest to regain health. He favoured the use of pituitrin and turpentine enemata.

Dr. Walter Swayne thought the more favourable modern results of treatment coincided with the introduction of aseptic technique.

Mr. Mumford remarked that an indication for drainage was the likelihood of a fæcal fistula forming. Washing out would be advantageous in cases where there was a wide extravasation of stomach contents.

Mr. Chitty considered the improvement in the results was largely due to early diagnosis and operation. He had had a series of 16 consecutive cases of perforated gastric or duodenal ulcer all operated on and the abdomen closed without drainage, and all had made good recoveries.

Mr. Moore mentioned a successful case of treatment for ascites in Banti's disease by drainage through the femoral canal into the thigh.

Mr. Temple said that in the period before surgeons operated for appendicitis he had, when making autopsies, several times found isolated fœcal concretions encapsuled in fibrous tissue in the peritoneal cavity, showing that in those days the peritoneum had looked after itself in some cases of appendicitis.

Mr. Fraser replied.

